# Epidural *versus* patient-controlled intravenous analgesia on pain relief and recovery after laparoscopic gastrectomy for gastric cancer: randomized clinical trial

**DOI:** 10.1093/bjsopen/zrad161

**Published:** 2024-01-18

**Authors:** Satoru Kikuchi, Takashi Matsusaki, Toshiharu Mitsuhashi, Shinji Kuroda, Hajime Kashima, Nobuo Takata, Ema Mitsui, Yoshihiko Kakiuchi, Kazuhiro Noma, Yuzo Umeda, Hiroshi Morimatsu, Toshiyoshi Fujiwara

**Affiliations:** Department of Gastroenterological Surgery, Okayama University Graduate School of Medicine, Dentistry and Pharmaceutical Sciences, Okayama, Japan; Department of Anesthesiology and Resuscitology, Okayama University Graduate School of Medicine, Dentistry and Pharmaceutical Sciences, Okayama, Japan; Center for Innovative Clinical Medicine, Okayama University Hospital, Okayama, Japan; Department of Gastroenterological Surgery, Okayama University Graduate School of Medicine, Dentistry and Pharmaceutical Sciences, Okayama, Japan; Department of Gastroenterological Surgery, Okayama University Graduate School of Medicine, Dentistry and Pharmaceutical Sciences, Okayama, Japan; Department of Gastroenterological Surgery, Okayama University Graduate School of Medicine, Dentistry and Pharmaceutical Sciences, Okayama, Japan; Department of Gastroenterological Surgery, Okayama University Graduate School of Medicine, Dentistry and Pharmaceutical Sciences, Okayama, Japan; Department of Gastroenterological Surgery, Okayama University Graduate School of Medicine, Dentistry and Pharmaceutical Sciences, Okayama, Japan; Department of Gastroenterological Surgery, Okayama University Graduate School of Medicine, Dentistry and Pharmaceutical Sciences, Okayama, Japan; Department of Gastroenterological Surgery, Okayama University Graduate School of Medicine, Dentistry and Pharmaceutical Sciences, Okayama, Japan; Department of Anesthesiology and Resuscitology, Okayama University Graduate School of Medicine, Dentistry and Pharmaceutical Sciences, Okayama, Japan; Department of Gastroenterological Surgery, Okayama University Graduate School of Medicine, Dentistry and Pharmaceutical Sciences, Okayama, Japan

## Abstract

**Background:**

Epidural analgesia (EDA) is a main modality for postoperative pain relief in major open abdominal surgery within the Enhanced Recovery After Surgery protocol. However, it remains unclear whether EDA is an imperative modality in laparoscopic gastrectomy (LG). This study examined non-inferiority of patient-controlled intravenous analgesia (PCIA) to EDA in terms of postoperative pain and recovery in patients who underwent LG.

**Methods:**

In this open-label, non-inferiority, parallel, individually randomized clinical trial, patients who underwent elective LG for gastric cancer were randomized 1:1 to receive either EDA or PCIA after surgery. The primary endpoint was pain score using the Numerical Rating Scale at rest 24 h after surgery, analysed both according to the intention-to-treat (ITT) principle and per protocol. The non-inferiority margin for pain score was set at 1. Secondary outcomes were postoperative parameters related to recovery and adverse events related to analgesia.

**Results:**

Between 3 July 2017 and 29 September 2020, 132 patients were randomized to receive either EDA (*n* = 66) or PCIA (*n* = 66). After exclusions, 64 patients were included in the EDA group and 65 patients in the PCIA group for the ITT analysis. Pain score at rest 24 h after surgery was 1.94 (s.d. 2.07) in the EDA group and 2.63 (s.d. 1.76) in the PCIA group (*P* = 0.043). PCIA was not non-inferior to EDA for the primary endpoint (difference 0.69, one side 95% c.i. 1.25, *P* = 0.184) in ITT analysis. Postoperative parameters related to recovery were similar between groups. More EDA patients (21 (32.8%) *versus* 1 (1.5%), *P* < 0.001) developed postoperative hypotension as an adverse event.

**Conclusions:**

PCIA was not non-inferior to EDA in terms of early-phase pain relief after LG.

Registration number: UMIN000027643 (https://www.umin.ac.jp/ctr/index-j.htm).

## Teaser

PCIA was not non-inferior to EDA in terms of early-phase pain relief after LG. Although PCIA did not impede recovery after LG, multimodal analgesia using alternatives for postoperative pain relief in addition to PCIA is required to improve the early-phase pain relief after LG.

## Introduction

Epidural analgesia (EDA) has been the main analgesia modality used after open abdominal surgery because it provides superior pain relief with fewer postoperative complications compared to opioid-based patient-controlled intravenous analgesia (PCIA)^1,2^. However, transitive haemodynamic instability due to sympathetic nerve blockage by EDA has been reported as an adverse event that can delay recovery after surgery^3–5^. Multimodal analgesia (MMA) has been recommended for postoperative pain management because it provides superior pain relief with minimal adverse events^6,7^. Therefore, whether EDA is an indispensable procedure for abdominal surgery remains controversial, especially when applied in minimally invasive surgery (MIS) such as laparoscopic procedures, which have shorter abdominal incisions, less postoperative pain and therefore faster recovery compared to open surgery^3,6–10^. Several prospective clinical trials have suggested that EDA delays recovery after surgery in laparoscopic colorectal surgery^3,4,11^.

In recent years, the use of MIS such as laparoscopic and robotic techniques has been increasing in gastric surgery, and its feasibility, safety and short- and long-term outcomes have been extensively confirmed^12–14^. Distal gastrectomy is a common surgical procedure for gastric cancer (GC), more than half of which are performed by MIS in Japan, and this proportion is increasing year by year^15^. However, the optimal pain management protocol after laparoscopic gastrectomy (LG) has not been established in the enhanced recovery after surgery (ERAS) group consensus guidelines^16^.

The aim of this RCT was to examine whether PCIA was non-inferior to EDA regarding pain score at rest 24 h after LG, and to compare the relative benefits of PCIA and EDA.

## Methods

### Study design

This was a single-centre prospective parallel group non-inferiority RCT with balanced randomization (1:1) that compared early-phase postoperative pain relief after LG and the relative benefits of fentanyl-based PCIA and EDA. The protocol was approved by the institutional review board (No. 1705-004) and all patients provided written informed consent before enrolment. This trial was registered with the University Hospital Medical Information Network Clinical Trial Registry (UMIN000027643) on 1 July 2017. The first patient was enrolled on 3 July 2017. The full study protocol was published at the start of the trial^17^ and is available in the *Supplementary material*.

### Patients

Patients scheduled for elective LG for GC at Okayama University Hospital in Japan and who met the eligibility criteria were enrolled in the study. Details of this study, as well as the inclusion and exclusion criteria, have been described previously^17^. Patients (20–80 years old) with an ASA Physical Status (ASA-PS) classification of I–III and scheduled for LG including total gastrectomy (TG), distal gastrectomy (DG) and proximal gastrectomy (PG) were included. The exclusion criteria were as follows: contraindication to placement of an epidural catheter; patients with an ongoing anticoagulant therapy; history of neoadjuvant chemotherapy; immunodeficiency; or palliative surgery.

### Randomization

Each patient was assessed at an outpatient consultation with their operating surgeon regarding fulfilling the eligibility criteria for participation in the study. All patients received verbal and written information about the study before written consent was obtained. The surgeons provided the research office with the necessary information for patient allocation after obtaining consent.

Patients were randomly assigned to the EDA or PCIA group (1:1) by a dedicated data manager in the research office using a computer-generated list in which allocation was performed by a stratified permuted block method on the day before surgery. Block sizes were 2 or 4. Whenever a new block was assigned, its size was determined randomly, adhering to a prespecified probability tailored to each stratum. In strata where the number of subjects was considered small, the probability of block size 2 was set high. Randomization was stratified by age (under or over 65 years old), sex (male or female), ASA-PS (1/2 or 3) and surgical procedure (DG or PG/TG) and the results of the allocation were provided to the surgeons by the research office. Surgeons who recruited patients were blinded for the randomization sequence. Blinding of patients and surgeons was not performed, as it was not realistically feasible for medical and logistic reasons. Allocated analgesia was provided to the patients and anaesthesiologists by the surgeons on the morning of surgery.

### Interventions, anaesthesia and pain management

The clinical pathway after LG in our institution was applied to all patients in order to standardize the perioperative treatment in both groups. In the EDA group, an epidural catheter was inserted at the thoracic level (Th 8–10) before induction of anaesthesia using the loss of resistance method. The epidural space was confirmed by the absorption test and a 3 ml bolus of 1% lidocaine with epinephrine (dilution 1:100 000). A 3–5 ml bolus of 0.2% ropivacaine was then injected and continuous infusion (2–4 ml/h) or bolus injection of 0.2% ropivacaine with fentanyl (1 µg/ml) was performed until the end of surgery depending on the anaesthesiologist’s decision, based on the patient’s vital signs. In the PCIA group, intravenous fentanyl and remifentanil were used as a pain management during surgery.

In both groups, induction of general anaesthesia was performed with propofol (target-controlled infusion [TCI] 2–4 µg/ml), remifentanil (0.2–0.5 µg/kg/min) and rocuronium bromide (0.5–0.6 mg/kg) for muscle paralysis. After tracheal intubation, anaesthesia was maintained with total intravenous anaesthesia technique using propofol TCI and remifentanil infusion, and rocuronium bromide as needed. All patients were extubated in the operating room.

Abdominal access was performed using the same technique in all patients. A 12-mm trocar was inserted through an umbilical incision and pneumoperitoneum was created. Four other trocars (one 12-mm and three 5-mm) were inserted in a rhombus position. After LG, resected specimens were extracted after extending the umbilical wound to 3–4 cm and intracorporeal reconstruction was performed after re-establishment of pneumoperitoneum using endoscopic linear stapler for DG and TG. Intracorporeal reconstruction by double-flap technique was performed for PG^18^.

Pain score was assessed by nursing staff twice daily (every 12 h) at rest and on mobilization, using the 0 (no pain) to 10 (worst pain imaginable) Numerical Rating Scale (NRS-11), which is a valid measure of pain intensity similar to the Visual Analogue Scale^19^. After surgery, EDA was maintained at a rate of 2–6 ml/h (target, NRS < 4), individually decided by the treating anaesthesiologist. Patients in the EDA group were allowed a bolus of 3 ml every 15 min. In the PCIA group, intravenous fentanyl (10 µg/ml) was started at 0–2 ml/h at the end of surgery (target, NRS < 4). A bolus of 1 or 2 ml was allowed every 15 min up to the maximum dose of 40 µg/h.

Both interventions were planned to be discontinued on postoperative day (POD) 2, but both could be continued until POD 7 if the anaesthesiologist judged that prolonged application would be beneficial for the patient. Treatment was judged ineffective by the anaesthesiologist when pain was not improved without haemodynamic change after administration of 10 ml of 1% lidocaine through the epidural tube in the EDA group, and increasing opioid doses increased adverse effects including nausea without pain relief in the PCIA group. All patients received celecoxib (200 mg × 2/day, oral) from POD 3 unless contraindicated. Additional analgesics including loxoprofen sodium hydrate (60 mg, oral), pentazocine (15 mg, intravenous), flurbiprofen axetil (50 mg, intravenous) and acetaminophen (1000 mg, intravenous) were administered as needed if patients complained of pain (NRS > 3) and those were allowed to be used at intervals of at least 4 h. The type and dose of all postoperative analgesics were recorded on the electronic medical record.

During their hospital stay, patients were monitored by nursing staff for adverse events related to both interventions, including sedation, postoperative nausea and vomiting (PONV), hypotension and urinary retention. Hypotension and PONV thought to be due to EDA or PCIA were treated by decreasing the baseline dose or stopping the infusion of analgesia depending on the anaesthesiologist’s decision.

### Endpoints and outcomes

Postoperative pain at rest 24 h after surgery was chosen as the primary endpoint, was assessed using NRS by nursing staff and analysed according to the intention-to treat (ITT) principle and per protocol (PP). Secondary endpoints were achievement of day of discharge criteria, the length of the postoperative hospital stay, postoperative complications, postoperative pain scores on days 2, 3, and 4 after surgery, additional doses of analgesics, adverse events related to analgesia and overall patient satisfaction. The discharge criteria were resuming a normal diet, no complaints of pain, full mobilization comparable to the patient’s preoperative status, and no unexpected adverse events for 24 h. These criteria were used to assess full recovery after surgery as achievement of discharge criteria, because the duration of the postoperative hospital stay is not only determined by medical factors but is influenced by social and logistic factors. Postoperative complications were graded using the Clavien–Dindo classification^20^. Postoperative pain was assessed by NRS from the evening of the surgery day until day 4 and it was assessed twice daily (morning and evening) from POD 1 to 4. Additional doses of analgesics, adverse events related to analgesia and patient satisfaction were recorded using a paper case report form. Patient satisfaction was graded from 1 (very dissatisfied) to 5 (very satisfied) using a 5-point Likert scale before the day of discharge. These were used to assess the effects and limitations of analgesia. All patients were followed up until a regular check-up at about one month after surgery. The primary endpoint was analysed according to the ITT principle (defined in the study protocol before study commencement) and PP (post-hoc). Secondary outcomes were all analysed according to the ITT principle.

### Statistical analysis

The sample size computation was based on retrospective data collected at our institution of patients undergoing LG for GC, with NRS scores at rest at 24 h after surgery being 2.11 (s.d. 1.9) in the EDA group and 2.36 (s.d. 1.8) in the PCIA group. Adopting a power of 80%, a one-side type I error (α) of 0.05, a non-inferiority margin of 1 in terms of NRS score and an anticipated drop-out rate of 5%, the calculated sample size was 62 patients per group^17^. The non-inferiority margin of 1 NRS point for pain was based on published minimal clinically important differences for acute pain of 1.65 points in the emergency department^21^.

The χ^2^ test was employed to analyse categorical variables. Welch’s *t*-test was used to compare continuous variables. All time point comparisons of the postoperative NRS were conducted using Welch’s *t*-test. To account for multiple comparisons, Holm’s method was applied for comparisons of all time points (secondary endpoints) except the NRS in the evening of postoperative day 1 (primary endpoint)^22^.

The primary endpoint was evaluated using a non-inferiority margin of 1 by one-side testing. Meanwhile, other endpoints underwent two-sided superiority evaluation. Values of *P* < 0.05 were considered significant (one-sided for the primary outcome and two-sided for all the other outcomes). Data were analysed using JMP 12.2 (SAS Institute, Cary, NC) and Stata 17/MP (Stata Crop LLC, College Station, TX, USA).

### Ethics statement

All procedures followed were in accordance with the ethical standards of the responsible committee on human experimentation (institutional and national) and with the Helsinki Declaration of 1964 and later versions.

## Results

**Fig. 1:**
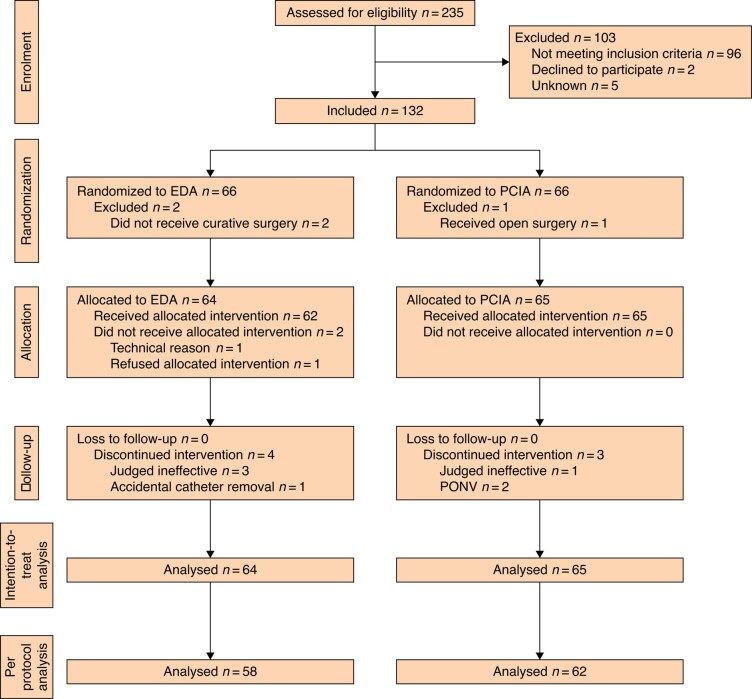
CONSORT flow diagram of the study

EDA, epidural analgesia; PCIA, patient-controlled intravenous analgesia; PONV, postoperative nausea and vomiting.

Between 3 July 2017 and 29 September 2020, 235 consecutive GC patients were assessed for eligibility. One hundred and three patients did not meet the inclusion criteria or refused to participate in the study. The remaining 132 patients were randomized to receive either EDA (*n* = 66) or PCIA (*n* = 66) as the allocated treatment. Three patients were excluded from the analysis for the following reasons: two EDA patients did not undergo curative resection due to peritoneal metastasis, and conversion from LG to open gastrectomy was necessary in one PCIA patient due to intraoperative bleeding. The final ITT analysis therefore compared 64 EDA patients with 65 PCIA patients (*Fig. 1*). The patients’ characteristics and demographics are listed in *Table 1*. Patient demographics, tumour stages and aspects of the surgical procedures were equally distributed between the groups. The surgery-related parameters are shown in *Table 2*. Although operation time and the amount of intraoperative blood loss were similar between the groups, operation room stay time was prolonged in the EDA group due to the time required for epidural catheter insertion. Intraoperative fentanyl and remifentanil use was increased in the PCIA group relative to the EDA group, because these were used as primary intraoperative pain management in PCIA without EDA. However, intraoperative infusion volume was slightly increased in the group using EDA for intraoperative anaesthesia management.

**Table 1:** Patient demographics and clinical characteristics

	EDA (*n* = 64)	PCIA (*n* = 65)
**Sex**		
Male	43 (67.2)	43 (66.2)
Female	21 (32.8)	22 (33.8)
Age (years), mean(s.d.)	66.7(10.2)	66.5(9.9)
BMI (kg/m^2^), mean(s.d.)	22.9(3.6)	22.5(3.0)
**ASA**		
I	19 (29.7)	23 (35.4)
II	43 (67.2)	39 (60.0)
III	2 (3.1)	3 (4.6)
ICU stay	0 (0)	1 (1.5)
Previous laparotomy	11 (17.2)	6 (9.2)
Stage*		
I	55 (85.9)	47 (72.3)
II	3 (4.7)	11 (16.9)
III	6 (9.4)	7 (10.8)
**Lymph node dissection**		
D1+	48 (75.0)	46 (70.8)
D2	16 (25.0)	19 (29.2)
**Surgical procedure**		
DG	47 (73.4)	48 (73.8)
PG	11 (17.2)	9 (13.8)
TG	6 (9.4)	8 (12.3)

Values are *n* (%) unless otherwise indicated. *Tumour stage is classified by Japanese Classification of Gastric Carcinoma, 3rd English edition. EDA, epidural analgesia; PCIA, patient-controlled intravenous analgesia; DG, distal gastrectomy; PG, proximal gastrectomy; TG, total gastrectomy.

**Table 2:** Surgical parameters

	EDA (*n* = 64)	PCIA (*n* = 65)	*P* (two-sided)
Operation time (min)	294(77.2)	281(69.1)	0.31
OR stay time (min)	383(77.1)	355(73.2)	0.04*
Blood loss (ml)	78.1(130.0)	65.5(100.5)	0.54
Intraoperative fentanyl use (µg)	164.1(120.7)	310.6(166.4)	<0.0001*
Intraoperative remifentanil use (µg)	2082(1302)	4066(1664)	<0.0001*
Intraoperative infusion volume (ml)	2666(794)	2409(658)	0.048*

Data are mean(s.d.). Statistical significance defined as *P < 0.05. EDA, epidural analgesia; PCIA, patient-controlled intravenous analgesia; OR, operation room.

### Duration of EDA and PCIA treatment after surgery

The doses of analgesia administered at baseline in both groups were determined by each treating anaesthesiologist. Therefore, 48 PCIA patients (73.8%) were maintained at 0 ml/h of fentanyl, whereas in contrast all EDA patients were maintained at 2–6 ml/h of 0.2% ropivacaine with fentanyl in the postoperative period. One patient did not receive EDA for technical reasons, and one EDA patient refused to undergo catheter insertion. Treatment was judged ineffective in three EDA patients and one PCIA patient and was discontinued before POD 2. Accidental catheter removal before POD 2 occurred in one EDA patient, and two PCIA patients discontinued before POD 2 due to PONV. These nine patients were excluded from the per protocol analysis, which included 58 patients in the EDA and 62 in the PCIA group (*Fig. 1*). Eight EDA and 10 PCIA patients discontinued before POD 2 because of adequate pain control and therefore requested discontinuation of treatment; however, all had the treatment for more than 24 h after surgery. EDA was left in place until POD 3 in two patients and until POD 6 in one patient. EDA and PCIA were discontinued according to the study protocol on POD 2 in 47 (73.4%) and 52 (80.0%) patients, respectively.

### Postoperative pain, surgical outcomes and recovery

In the ITT analysis, postoperative pain score in the EDA group in the evening of POD 1, which was the primary endpoint of this study, was 1.94 (s.d. 2.07), which was significantly lower than that in the PCIA group (2.63 (s.d. 1.76), *P* = 0. 043). As a consequence, the difference of 0.69 and the resulting one side 95% c.i. of 1.25 crossed the non-inferiority margin of 1 and did not show the non-inferiority of PCIA (*P* = 0.184) for pain control on the evening of POD 1 compared to EDA in ITT analysis (*Table 3*). Similarly, in the PP analysis, pain score at 24 h after surgery was lower in the EDA group (1.7, s.d. 1.9) compared to the PCIA group (2.7, s.d. 1.8, *P* = 0. 005). The difference of 0.93 (one side 95% c.i.: 1.49) also did not show the non-inferiority of PCIA in the PP analysis (*P* = 0.424).

**Table 3:** Postoperative outcomes in each group

	EDA (*n* = 64)	PCIA (*n* = 65)	Difference (95% c.i.)	*P*	
				Non-inferiority (one-sided)	Superiority† (two-sided)
Pain score at postop 24 h	1.9(2.1)	2.6(1.8)	0.69 (1.25)	0.184	
Achievement of discharge criteria (days)	9.9(5.7)	9.7(5.5)	−0.10 (−2.05,1.84)		0.915
Postoperative hospital stay (days)	12.1(5.6)	12.2(5.5)	0.01 (−1.91,1.93)		0.989
Additional doses of analgesics‡	2.8(3.4)	2.3(2.3)	−0.60 (−1.54,0.47)		0.293
Postoperative complication, *n* (%)	11 (17.2)	12 (18.5)	1.27% (−11.9%,14.4%)		0.850
Patients’ satisfaction§	3.9(1.2)	4.1(1.0)	0.26 (−0.18,0.69)		0.249
First day of flatus (days)	2.0(1.0)	2.6(1.0)	0.63 (0.28,0.98)		< 0.001*
Fully mobilization day (days)	1.6(0.7)	1.5(0.7)	−0.16 (−0.41,0.08)		0.191

Values are mean(s.d) unless otherwise indicated. Statistical significance defined as *P < 0.05. †Equality test: H0 ‘difference = 0’, Ha: ‘difference ≠ 0’. ‡The doses of additional analgesics were reviewed using the electronic medical record. §Seven missing in the PCIA group and 18 in the EDA group. EDA, epidural analgesia; PCIA, patient-controlled intravenous analgesia.

**Fig. 2:**
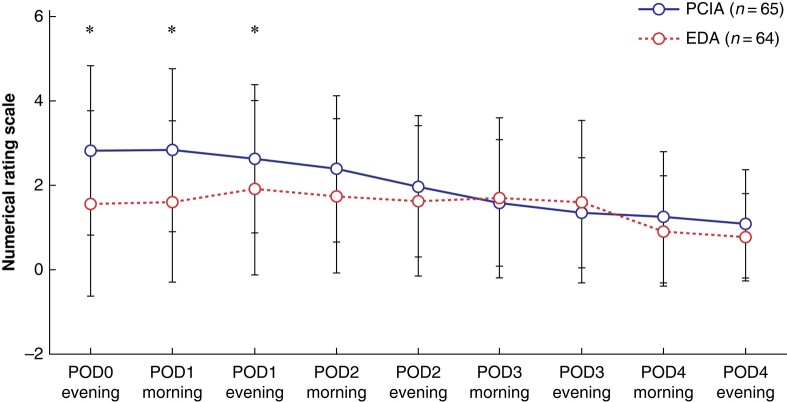
Postoperative pain scores in the intention-to-treat analysis

Regarding the further time points (secondary endpoints), the postoperative NRS score was significantly lower in the EDA group in the evening of the day of surgery (1.57 (s.d. 2.19) *versus* 2.83 (s.d. 2.00), *P* = 0.001) and in the morning of POD 1 (1.62 (s.d. 1.90) *versus* 2.83 (s.d. 1.93), *P* = 0.001) compared to the PCIA group after adjusting for multiple testing using Holm’s method (*Fig. 2*). Pain score from POD 2 onwards was comparable between groups.

Data are presented as mean(s.d.). Pain was evaluated at rest using an NRS ranging from 0 to 10, on the evening after surgery, and twice a day thereafter until POD 4 for EDA patients (red circles) and PCIA patients (blue circles). All time-point comparisons of the postoperative numerical rating scale were conducted using Welch’s *t*-test. To account for multiple comparisons, Holm’s method was applied for all time points except the primary endpoint (POD 1 evening). *Indicates significant difference. POD, postoperative day; EDA, epidural analgesia; PCIA, patient-controlled intravenous analgesia.

In terms of recovery after surgery, assessed secondary outcomes such as achievement day of discharge criteria and duration of postoperative hospital stay were similar between the groups. Day of full mobilization was also similar between the groups, although first day of flatus was significantly sooner in the EDA group. Furthermore, postoperative complications (≥C–D grade 2), additional doses of analgesics and patient satisfaction were comparable between the groups (*Table 3*).

### Adverse events related to analgesic procedures

There were no severe complications related to EDA placement such as epidural haematoma, abscess or nerve injury. Significantly more EDA patients developed postoperative hypotension with symptoms such as dizziness, headache and palpitations compared to the PCIA group (32.8% *versus* 1.5%, *P* < 0.001), although no patient required any vasopressor administration for hypotension (*Table 4*). There were no significant differences in regard to postoperative nausea and vomiting or urinary retention (*Table 4*).

**Table 4:** Adverse events related to the analgesic procedures

	EDA (*n* = 64)	PCIA (*n* = 65)	*P* (two-sided)
**PONV**			
0	55 (85.9)	55 (84.6)	0.379
1	9 (14.1)	8 (12.3)	
2	0 (0)	2 (3.1)	
Hypotension	21 (32.8)	1 (1.5)	<0.001*
Urinary retention	0	0	N/A

Values in parentheses are percentages. Statistical significance defined as *P < 0.05. EDA, epidural analgesia; PCIA, patient-controlled intravenous analgesia; PONV, postoperative nausea and vomiting; N/A, not applicable.

## Discussion

This is the first RCT to assess the effect of pain control with PCIA compared to EDA in the early phase after surgery in LG for GC. The results of the present study could not confirm that PCIA was non-inferior to EDA in terms of pain relief at 24 h following LG in both the ITT and PP analysis. Furthermore, PCIA patients had slightly elevated NRS pain scores in the first 24 h after the operation. However, the similarity of pain scores from POD 2 onwards, the achievement day of discharge criteria, the equal overall duration of postoperative hospital stay and comparable postoperative complication rates between the groups suggests that PCIA does not impede recovery after LG. Although PCIA was shown to be not non-inferior to EDA, postoperative pain scores in both groups (EDA and PCIA) were generally low (<3) and differences small.

In the present study, pain management was judged ineffective in only three EDA patients (4.8%), which is very low. Although the success rate of EDA procedures depends on the institutional technique, failure rates are more frequent for EDA than other techniques and have been reported as up to 30% in clinical practice^3,23^. The high success rate of EDA can be attributed to its superior pain management in the early phase after surgery compared with PCIA, as found in the present study. Intravenous fentanyl doses at baseline were determined by each treating anaesthesiologist. Forty-eight PCIA patients (73.8%) were therefore maintained at 0 ml/h of fentanyl direct following surgery because of the great concern of many anaesthesiologists regarding the occurrence of postoperative respiratory depression by fentanyl in the general ward, which might be responsible for the slightly poorer pain management in the PCIA group.

MMA is designed to provide enhanced analgesic effect by combining analgesics with different mechanisms and has recently been recommended for abdominal surgery in the ERAS consensus statement because MMA reduces opioid consumption, minimizes adverse events caused by analgesics, improves pain relief, enhances earlier recovery and reduces medical costs^6,7^. Some prospective RCTs have demonstrated that postoperative routine administration of intravenous acetaminophen improved pain relief after gastrectomy for GC and several other laparoscopic surgeries^24,25^. In the present study, PCIA was inferior to EDA in terms of postoperative pain relief within 24 h after surgery.

In terms of postoperative recovery, several parameters were similar between the groups, including postoperative hospital stay and achievement day of discharge criteria, although first day of flatus was significantly sooner in the EDA group. A previous study has reported that EDA enhanced the recovery of bowel motility after LG due to regional sympathectomy to the gastrointestinal tract^26^. However, it is unclear whether recovery of bowel motility is synonymous to recovery after surgery. Although hospital stay relies on various factors, including the health insurance system in each country, achievement of discharge criteria is also influenced by local centre-specific attitudes. Most patients who undergo gastrectomy gradually resume oral intake by liquid diet or soft meals after surgery from POD 2 or 3 onward to avoid post-gastrectomy syndrome and in fear of anastomotic leakage, although no trial has reported increased adverse outcomes from early introduction of oral food intake for patients undergoing upper gastrointestinal surgery^27–29^. After gastrectomy, most patients resume eating a normal diet at POD 5–7, depending on the clinical pathway of the institution. Therefore, the slightly shorter time to recovery of bowel motility with EDA does not come to advantage in terms of postoperative hospital stay or achievement of discharge criteria. In contrast, more than 30% of EDA patients developed postoperative temporary hypotension, which was significantly higher than in the PCIA group. Postoperative temporary haemodynamic instability due to thoracic sympathetic nerve blockage in EDA patients can hinder postoperative mobilization and recovery, and has been reported in several studies although the number of days until full mobilization in both groups were similar in the present study^3,5,30^.

Regarding intraoperative parameters related to general anaesthetic management, intraoperative infusion volume was higher in the EDA group than in the PCIA group, whereas intraoperative opioid use was significantly lower, given that EDA was used for intraoperative pain relief whereas PCIA was started at the end of surgery. The ERAS guidelines for gastrectomy recommend avoiding electrolyte and water overload in the perioperative period to reduce postoperative complications^12,31,32^. PCIA might be advantageous in terms of minimizing intraoperative infusion volume. Furthermore, operation room (OR) stay time was significantly longer in the EDA group than in the PCIA group, whereas operation time in turn was similar. EDA takes longer because of the time required for the anaesthesiologist to insert the epidural catheter. The longer OR stay time associated with EDA might translate into increased total medical costs due to facility and medical personnel costs; however, examination of the financial aspect associated with the two compared procedures was not part of the current study.

The ERAS guidelines include multimodal perioperative care, are based on evidence and aim to reduce adverse sequelae of surgical stress, accelerate recovery and reduce postoperative complications and total costs^33^. EDA is a key item of ERAS because it provides superior intra- and early postoperative pain relief and decreases the rates of pneumonia in open abdominal surgery^1,2^. However, the effects of EDA on postoperative recovery are little examined in LG. In fact, more than 50% of institutions have not applied EDA for MIS gastrectomy^34^. This is probably because the ERAS guidelines are intended as medical guidelines rather than specific rules and there are no fixed conditions regarding their implementation. These are modified periodically in response to new evidence and changes in clinical practice.

There are several limitations in the current study. First, medical staff including anaesthesiologists, surgeons and nurses, as well as the patients, were not blinded to the analgesic methods. Second, the basal infusion rate of analgesia was decided in all cases by the treating anaesthesiologist, which could have affected the postoperative outcomes. However, this situation also reflects clinical reality.

In summary, PCIA was not non-inferior to EDA in terms of early-phase pain relief after LG; however, it still provided adequate pain relief after LG and the overall postoperative course was comparable between the two interventions.

## Supplementary Material

zrad161_Supplementary_Data

## Data Availability

Data access and the full study protocol are available from the corresponding author upon reasonable request.
