# Supplementary material for: Epidural versus patient-controlled intravenous analgesia on pain relief and recovery after laparoscopic gastrectomy for gastric cancer: randomized clinical trial
Source: BJS Open. 2024 Jan 18;8(1):zrad161. doi: 10.1093/bjsopen/zrad161 (PMC10798823; doi:10.1093/bjsopen/zrad161)
Supplement: zrad161_Supplementary_Data [file zrad161_supplementary_data.docx]

**Epidural versus patient-controlled intravenous analgesia on pain relief and recovery after laparoscopic gastrectomy for gastric cancer: Randomized clinical trial**

Satoru Kikuchi^1*^, Takashi Matsusaki^2^, Toshiharu Mitsuhashi^3^, Shinji Kuroda^1^, Hajime Kashima^1^, Nobuo Takata^1^, Ema Mitsui^1^, Yoshihiko Kakiuchi^1^, Kazuhiro Noma^1^, Yuzo Umeda^1^, Hiroshi Morimatsu^2^, and Toshiyoshi Fujiwara^1^

Department of ^1^Gastroenterological Surgery, and ^2^Anesthesiology and Resuscitology, Okayama University Graduate School of Medicine, Dentistry and Pharmaceutical Sciences, Okayama 700-8558, Japan

^3^Center for Innovative Clinical Medicine, Okayama University Hospital, Okayama 700-8558, Japan

**Corresponding author.** Satoru Kikuchi, Department of Gastroenterological Surgery, Okayama University Graduate School of Medicine, Dentistry, and Pharmaceutical Sciences, 2-5-1 Shikata-cho, Kita-ku, Okayama 700-8558, Japan

Tel: +81-86-235-7255; Fax: +81-86-221-8775; E-mail: satorukc@okayama-u.ac.jp **ORCID ID**; 0000-0002-7671-0696

**Supplementary Materials - Index**

| **Supplementary Methods** |  |
| --- | --- |
| Study Protocol | *page 2-14* |

**Supplementary Methods**

**Study Protocol.**

**A prospective randomized controlled clinical trial of the analgesic efficacy of postoperative epidural analgesia (EDA) versus intravenous patient-controlled analgesia (iv-PCA) in laparoscopic gastrectomy.**

UMIN registration number: UMIN000027643

**Principal investigator**

Toshiyoshi Fujiwara

Okayama University Graduate School of Medicine, Dentistry and Pharmaceutical Sciences

Department of Gastroenterological Surgery

2-5-1, Shikata-cho, Kita-ku, Okayama City, Okayama Prefecture, 700-8558

Tel: +81-86-235-7257

Fax: +81-86-221-8775.

E-mail: toshi_f@md.okayama-u.ac.jp

**Research Secretariat**

Satoru Kikuchi

Okayama University Graduate School of Medicine, Dentistry and Pharmaceutical Sciences

Department of Gastroenterological Surgery

2-5-1, Shikata-cho, Kita-ku, Okayama City, Okayama Prefecture, 700-8558

Tel: +81-86-235-7257

Fax: +81-86-221-8775

E-mail: satorukc@okayama-u.ac.jp

13 April 2020 Third edition.

**0. Overview.**

**0.1. objectives**

　To test the non-inferiority of intravenous patient-controlled analgesia (iv-PCA) as postoperative analgesia to epidural analgesia (EDA) with respect to post-operative analgesia in patients aged 20 years and older undergoing laparoscopic gastrectomy for gastric cancer.

● Primary endpoint

　　　　　　Pain score using Numerical Rating Scale (NRS) at rest 24 hours after surgery.

　　● Secondary endpoints

　　　　　　Days to achieve discharge criteria*, Duration of postoperative hospital stay, Incidence of postoperative complications, Pain score (NRS) on postoperative days 2, 3 and 4 at rest, Additional analgesic usage, Analgesic method-related complications.

*Discharge criteria: (1) Good pain control with oral analgesics (2) Able to walk on own (3) Consume more than half of meal (4) No post-operative complications, or as far as possible treated on an outpatient basis.

● Safety assessment.

　　　　　　Frequency of analgesia-related adverse events.

**0.2. subject.**

　Inclusion criteria.

1) Patients with gastric cancer which has been diagnosed histologically and scheduled to undergo laparoscopic gastrectomy (total gastrectomy, distal gastrectomy or proximal gastrectomy).

　　　2) Patients aged between 20 and 80 years

　　　3) Patients who can provide written informed consent

　Exclusion criteria.

1) Patients with a preoperative ASA-PS (American Society of Anesthesiologists Physical Status) of 4 or higher

　　　2) Patients undergoing anticoagulant therapy

　　　3) Medical contraindication for EDA

4) Abnormal anatomy of the spinal column

5) Patients undergoing neoadjuvant chemotherapy

6) Patients with immunodeficiency

7) Patients undergoing palliative surgery

8) Patients undergoing emergency surgery

　　　9) Otherwise judged by the investigator as unsuitable for enrollment

**0.3. analgesic methods**

　　　Epidural analgesia (EDA: Epidural Analgesia).

　　　　0.2% ropivacaine + fentanyl (1 µg/mL) continuous, 2-6 ml/hr, bolus 3 ml, 15 min intervals

　　　Intravenous-patient-controlled analgesia 　　　(iv-PCA: intravenous patient-controlled analgesia).

　　　　Fentanyl (10 µg/mL) continuous, 0-2 ml/h, 1-2 ml bolus, 15 min intervals, max 4 doses/hr

**0.4. expected enrolment and study duration**

　　　Expected number of patients enrolled: 132 (66 in each group)

　　　Registration period : 3 years and 6 months (from 1 July 2017 to 31 December 2020)

　　　Analysis period. : 6 months.

　　　Total study period : 4 years (from 1 July 2017 to 30 June 2021)

**0.5. contact details.**

　　　Principal investigator: Toshiyoshi Fujiwara

Graduate School of Medical and Dental Sciences, Okayama University

Department of Gastroenterological Surgery

2-5-1, Shikata-cho, Kita-ku, Okayama City, Okayama Prefecture, 700-8558

E-mail: toshi_f@md.okayama-u.ac.jp

　　　Research Secretariat: Satoru Kikuchi

Department of Gastrointestinal Surgery, Okayama University Hospital

2-5-1, Shikata-cho, Kita-ku, Okayama City, Okayama Prefecture, 700-8558

E-mail: satorukc@okayama-u.ac.jp

**1. background**

**epidural analgesia as postoperative analgesia**

　　　In abdominal surgery, epidural anaesthesia, used intraoperatively alone or in combination with general anaesthesia, has been reported to have many benefits in terms of intraoperative management. It has also been reported to have many advantages in postoperative analgesia, such as reducing postoperative respiratory complications and preventing the occurrence of ileus and has been established as an essential method of analgesia in abdominal surgery.

　　　On the other hand, however, epidural analgesia may carry the risk of serious complications such as hematomas, abscesses, and nerve damage. The frequency of serious complications is considered to be extremely rare and has been reported to be around 1 in 150,000 to 190,000 cases. Various biases have been shown to play a significant role in their frequency, and the choice of epidural analgesia should be considered the risks from multiple perspectives.

**increased use of laparoscopic surgery for gastric cancer**

In recent years, laparoscopic surgery has become increasingly popular in abdominal surgery. In gastric cancer surgery, a 2013 national survey by the Japanese Society for Endoscopic Surgery revealed that more than 42% of gastric cancer operations were performed laparoscopically, and the proportion is increasing. The minimally invasive nature of laparoscopic surgery compared to open surgery has been reported in many cases, showing its superiority in terms of cosmetics, reduced wound pain, enhanced postoperative respiratory function, reduced postoperative hospital stay and early recovery.

**epidural analgesia for laparoscopic colorectal resection**

　　　The Enhanced recovery after surgery (ERAS) protocol is a multidisciplinary treatment programme that aims to achieve rapid recovery after highly invasive surgery by integrating a range of evidence-based treatments to help accelerate recovery after surgery. ERAS protocols have been proven to reduce postoperative complications, hospital stays and healthcare costs after colorectal resection. Within the ERAS protocols, fluid management, minimally invasive surgery and epidural analgesia are considered. However, the effectiveness of epidural analgesia is debatable in minimally invasive surgery such as laparoscopic colorectal surgery, with recent reports suggesting that epidural analgesia may even delay postoperative recovery. The ERAS Group guidelines also state that epidural analgesia and patient-controlled analgesia (PCA) are equivalent in laparoscopic colorectal resection.

**subject.**

　　　The incidence of gastric cancer, the subject of this study (2011 statistics), is still the leading malignant neoplasm in Japan, ranking first among men and third among women, and surgery is the first choice for the treatment of resectable lesions. Intraoperative and postoperative analgesia for gastric cancer surgery, including laparoscopic surgery, is currently the standard method of analgesia, unless epidural analgesia is contraindicated, such as when anticoagulants are being administered.

**significance**

　　　Epidural analgesia is now routinely used in intra- and postoperative analgesia for abdominal surgery and has proven useful as postoperative analgesia. However, epidural analgesia may be associated with the risk of serious complications such as haematoma, abscess and nerve damage. Furthermore, adverse events such as postoperative hypotension and urinary retention have also been reported with epidural analgesia, which, while effective for pain relief, may lead to delayed postoperative mobilizaion and may not lead to a true early postoperative recovery.

　　　Furthermore, recent advances in echo-guided peripheral nerve blocks other than epidural analgesia, intravenous patient-controlled analgesia (iv-PCA) and multimodal analgesia (a combination of various analgesia), in addition to the widespread use of laparoscopic surgery for gastric cancer, have made the need for epidural analgesia for epidural analgesia is debatable. If iv-PCA proves to be non-inferior to epidural analgesia (EDA) in postoperative pain relief for laparoscopic gastrectomy, it could provide safer and simpler postoperative analgesia and reduce the burden and healthcare costs for gastric cancer surgery patients.

**2. research methods**

**types and designs of research**

　　　Prospective randomised controlled clinical trial of early recovery with postoperative epidural analgesia (EDA) versus patient-controlled analgesia (iv-PCA) in patients undergoing laparoscopic gastrectomy for gastric cancer.

**the expected duration of the research subject's participation in the study**

The period of study participation is from the time consent is obtained in the preoperative period until discharge from hospital after surgery.

**Intervention**

Use epidural analgesia (EDA) or patient-controlled analgesia (iv-PCA) as postoperative analgesia. Dosages are equivalent to those in routine practice.

● Epidural analgesia (EDA)

A catheter is inserted into the thoracic spine (Th8-Th10) epidural space before general anaesthesia and 0.2% ropivacaine + fentanyl (1 µg/mL) is administered continuously, 2-6 ml/hr, 3 ml bolus, every 15 min, starting postoperatively, in principle until the second postoperative day, but can be continued until the seventh postoperative day if the pain management team considers it advisable.

● Intravenous patient-controlled Analgesia (iv-PCA)

　　Fentanyl (10 µg/mL) administered intravenously continuously (0-2 ml/h, 1-2 ml bolus, 15 min intervals, max 4 doses/h) from postoperative period, in principle until the second postoperative day, but can be continued until the seventh postoperative day if the pain management team considers it advisable.

**equipment and materials used for the intervention.**

　　　Use epidural catheter kit for epidural catheter insertion.

　　　Use 0.2% ropivacaine (Anapain®) and fentanyl injection as epidural drugs.

　　　Fentanyl injection solution is used for intravenous patient-controlled analgesia.

　　　Both epidural and patient-controlled analgesia are administered using a PCA pump.

**provisions on concomitant medications.**

　　　Concomitant medications: acetaminophen (Acerio®) Used as additional analgesia in cases of poor pain relief (NRS≥3) during postoperative epidural analgesia or patient -controlled analgesia, 1000 mg per dose over 15 minutes with a minimum interval of 4 hours between doses. However, it should not be administered to patients with contraindications to paracetamol administration. For patients weighing less than 50 kg, a maximum dose of 15 mg per kg of body weight should be administered. Mainly used up to the third postoperative day before oral intake is started.

　　　　　　　Celecoxib (Celecox®) Two tablets (200 mg) twice daily, after breakfast and dinner, with oral intake starting on the third postoperative day, to be continued until the day of discharge. It can be discontinued before discharge if the patient wishes to discontinue and is not administered to patients with contraindications to celecoxib administration.

　　　　　　　Flurbiprofen (Ropion®) Additional analgesia in case of poor pain (NRS≥3) with postoperative epidural analgesia or patient-controlled analgesia 50 mg per dose diluted in 100 ml of saline and administered over 30 minutes with a minimum interval of 4 hours between doses. It is mainly used up to the third postoperative day before oral intake is started.

　　　　　　　Loxoprofen (Loxonin®) From postoperative day 3 after oral intake has started, as an additional analgesic in case of poor pain relief (NRS≥3) with epidural or patient-controlled analgesia One tablet (60 mg) should be taken orally at least 4 hours between doses.

　　　　　　　Pentazocine (Sosegon®) Used as additional analgesia in cases of poor pain relief (NRS≥3) with postoperative epidural analgesia or patient-controlled analgesia; 15 mg per dose diluted in 100 ml of saline and administered over 30 minutes with a minimum interval of 4 hours between doses. Mainly used up to the third postoperative day before oral intake is started.

**methods of cessation (withdrawal)**

If neurological symptoms or pain or swelling at the site of epidural catheter insertion (see Adverse events for definitions) are observed, the epidural analgesia should be discontinued. Epidural analgesia should also be discontinued if self-extraction of the epidural catheter by the patient occurs postoperatively. Both epidural analgesia and patient-controlled analgesia should be discontinued if adverse events such as nausea caused by fentanyl are difficult to control.

**research subject guidance information**

　　　Patients are instructed in the use of the PCA and PCEA pumps by the anaesthetist in charge and the nurse in charge in the post-operative recovery room and after returning to the ward.

**case registration and allocation methods**

How to enrol research subjects: the principal investigator or research associate 1) obtains written consent; 2) The date consent was obtained and the information necessary to correspond the subject to the subject identification code should be noted on the list of subject identification codes kept by the principal investigator; 3) Submit the case registration form with the subject identification code to the research office 4) Receive confirmation of eligibility and a registration confirmation form with the subject registration number and the analgesic method assigned. 5) Report promptly when there is withdrawal of consent, discontinuation, drop-out, etc.

　Method of allocation of study subjects: allocation of study subjects to each treatment group is performed by the dedicated data manager. The subjects will be allocated to each treatment group in the order of case registration according to a pre-designed allocation list using the stratified substitution block method, and a confirmation of registration will be issued, indicating the subject registration number and the name of the treatment group. The allocation table is kept by the secretariat.

**actions to be taken after the completion of the research.**

After the completion of this study, the doctor in charge will provide the medical care he or she considers most appropriate for the study subjects, including the results obtained in this study.

**3. Observations and laboratory tests**

**pre-registration assessment items (pre-operative)**

1) Medical history interview:

　　　　　History of spinal compression fractures

　　　　　On anticoagulant/antiplatelet drugs

　　　　　Presence or absence of back infection.

　　　　　General condition: PS(ECOG), ASA

Age.

　　　　　Height, weight.

　　　　　Respiratory failure.

　　　　　Cancer Chemotherapy

　　　　　Severe infections.

　　　　　Drug allergy.

　　　　　co-morbidities

　　　2) 12-lead ECG at rest

　　　3) Respiratory function tests: %VC, FEV 1.0%.

　　　4) Blood tests: white blood cells, red blood cells, haemoglobin, platelets

　　　5) Biochemical tests: albumin, total bilirubin, AST, ALT, BUN, creatinine

　　　6) Coagulation tests: PT, APTT, D-dimer

　　　7) Simple chest X-ray

　　　8) CT scan of the abdominopelvic region (simple CT is acceptable if contrast-enhanced CT is not possible due to contrast allergy).

　　　9) Upper gastrointestinal endoscopy (with diagnosis of gastric cancer on histopathological examination)

**surgical and tumour endpoints**

　　　1) Surgical technique (resection method, reconstruction method, degree of lymph node dissection, presence or absence of complications of resected organs).

2) Surgical time

　　　3) Blood loss and transfusion volume

　　　4) Pathological findings of the tumour (histological type, wall depth, lymph node metastasis, histological progression and overall radiculopathy)

**post-operative endpoints (until discharge)**

Basically, it conforms to the post-operative clinical pathway for gastric cancer currently in use at the hospital.

　　　1) Check vital signs: blood pressure, pulse, temperature, oxygen saturation

　　　2) Blood tests (postoperative days 1, 3 or 4 and 7)

White blood cells, red blood cells, haemoglobin, platelets

　　　3) Biochemical tests (postoperative days 1, 3 or 4 and 7)

Albumin, total bilirubin, AST, ALT, BUN, creatinine, CRP

　　　4) Coagulation test (postoperative day 7)

PT, APTT, D-dimer.

　　　5) Pain assessment

Pain scores at rest and during body movement are assessed twice daily according to the Numerical Rating Scale: NRS.

　　　6) Additional analgesic usage

　　　　　　Additional analgesic

　　　7) Patient satisfaction.

**post-operative endpoints (one month after discharge)**

　　　Interview and examine the patient for the development of analgesia-related adverse events at the time of the outpatient visit.

　　　If it is difficult to come to the hospital within one month after surgery, it is acceptable to come to the hospital within three months after surgery.

**adverse events/side effects**

**adverse events**

Any new unfavourable or unintended signs (including abnormal laboratory values), symptoms, diseases or events that occur after the start of the study, whether or not causally related to epidural analgesia and patient-controlled analgesia.

**side effects**

An adverse event is defined as any event that is determined, for some reasonable reason, to be associated with the epidural analgesia or patient-controlled analgesia procedure. In other words, an adverse event is defined as any medically determined adverse event that occurs in a subject because of the epidural analgesia or patient-controlled analgesia procedure. Generally, an association with epidural analgesia or patient self-administered analgesia is assessed as 'yes' when the rationale or reason for the association can be identified.

**serious adverse events or serious side effects**

Among adverse events, serious adverse events are defined as the following.

(i) Deceased.

(ii) Of life-threatening substances.

(iii) Those requiring hospitalisation or an extended period of hospitalisation for treatment.

(iv) Permanent or marked impairment or dysfunction.

(v) those with congenital diseases or anomalies in later generations.

**recording and assessment of adverse events**

For adverse events, the following criteria are used to assess severity and causal relationship to epidural analgesia or patient-controlled analgesia. The severity and causal relationship assessment will be determined in consultation with the Effectiveness and Safety Assessment Committee*.

　　　*The Effectiveness and Safety Assessment Committee shall consist of the following three members.

　　　　Assistant Professor Shunsuke Tanabe, Department of Gastrointestinal Surgery, Okayama University Hospital

　　　 Assistant Professor Kazuhiro Yoshida, Department of Hepatobiliary and Pancreatic Surgery, Okayama University Hospital

　　　　Third-party position Assistant Professor Takayuki Iwamoto, Center for Innovative Clinical Medicine, Okayama University Hospital

**severity assessment**

Evaluate the severity of adverse events/side effects according to CTCAE v4.0.

**causality assessment**

[Clearly relevant] : clearly related to epidural analgesia or patient self-administered analgesia from a clinical/biological point of view. In this case, treat as a side effect.

[Probably related]: strongly suspected to be related to epidural analgesia or patient self-administered analgesia from a clinical/biological point of view and for which a reasonable explanation is possible, such as time course from epidural analgesia or patient self-administered analgesia use to the occurrence of the adverse event or its disappearance after discontinuation of use. In such cases, treat as an adverse effect.

[Likely association]: suspected association with epidural analgesia or patient self-administered analgesia from a clinical/biological point of view, based on the time course from the start of epidural analgesia or patient self-administered analgesia use to the occurrence of the adverse event. In this case, treat as an adverse effect.

[Probably not related]: when an association with epidural analgesia or patient self-administered analgesia can be ruled out and other causes are indicated. In this case, treat as an adverse event.

[Obviously not related]: when an association with epidural analgesia or patient self-administered analgesia can be clearly ruled out and a link to other causes is strongly considered. In this case, treat as an adverse event.

**4. Evaluation items**

　　　● Primary endpoint

　　　　　　Pain score using Numerical Rating Scale (NRS) at rest 24 hours after surgery.

　　 ● Secondary endpoints

　　　　　　Days to achieve discharge criteria*, Duration of postoperative hospital stay, Incidence of postoperative complications, Pain score (NRS) on postoperative days 2, 3 and 4 at rest, Additional analgesic usage, Analgesic method-related complications.

*Discharge criteria: (1) Good pain control with oral analgesics (2) Able to walk on own (3) Consume more than half of meal (4) No post-operative complications, or as far as possible treated on an outpatient basis.

**5. Data aggregation and statistical analysis methods**

**data aggregation**

　　　The types of reports to be submitted are listed below, and the Secretariat will enter information on the submitted reports into a database.

　　　Case registration form ............................. Submitted to the Secretariat at the time of registration.

　　　Case record form (pre-surgery) .................... Submitted to the office before surgery.

　　　Case record form (during hospitalisation) .................... Submitted to the secretariat as soon as possible after discharge from hospital.

　　　Adverse event report form ........................... Submitted to the Secretariat as soon as possible upon occurrence of an adverse event.

**statistical analysis methods**

　　　After enrolment in the study, patients who did not withdraw their consent and for whom protocol treatment was administered will be included in the analysis population (Full analysis set, FAS). For the analysis of the primary endpoint, NRS at 24hrs after surgery of the EDA and iv-PCA groups will be calculated for the FAS and analysed by Student t-test. Secondary endpoints will be analysed by the X^2^ test was employed to analyze categorical variables. Welch’s t test was used to compare continuous variables.

**6. Target case numbers and rationale for setting them**

[Target number of cases] 132 cases

[Rationale for setting up the case number].

In a retrospective study of patients who underwent laparoscopic gastrectomy for gastric cancer at our hospital and who received postoperative pain management with EDA or iv-PCA, respectively, the mean 24-hour postoperative pain score (NRS) was 2.11 and standard deviation 1.9 in the EDA group, and in the iv-PCA group the mean 2.36 with a standard deviation of 1.8.

Since only patients who meet the inclusion criteria and do not meet the exclusion criteria will be included in this study, it is assumed that the variation in scores will be smaller than in the existing data, as the patients will be more similar than in the existing data. Therefore, in the caseload design of this study, it is assumed that the standard deviation will be about 10% smaller, with a mean of 2.11 and a standard deviation of 1.71 for the EDA group and a mean of 2.36 and a standard deviation of 1.62 for the iv-PCA group.

Assuming a non-inferiority margin of 1 and a sample size of α = 0.05 and β = 0.2, the sample size is 62 cases in one group (124 cases in both groups). Assuming a drop-out rate of approximately 5%, the target number of cases is 132 in both groups.

**7. Duration of the study**

From 1 July 2017 to 30 June 2021

(Deadline for registration: 31 December 2020)

**8. Policy for establishing research subjects**

Study subjects who meet all of the following selection criteria and none of the exclusion criteria are eligible cases for study enrolment.

**Inclusion criteria**

1) Patients with gastric cancer which has been diagnosed histologically and scheduled to undergo laparoscopic gastrectomy (total gastrectomy, distal gastrectomy or proximal gastrectomy).

　　　2) Patients aged between 20 and 80 years

　　　3) Patients who can provide written informed consent

**Exclusion criteria**

　　　1) Patients with a preoperative ASA-PS (American Society of Anesthesiologists Physical Status) of 4 or higher

　　　2) Patients undergoing anticoagulant therapy

　　　3) Medical contraindication for EDA

4) Abnormal anatomy of the spinal column

5) Patients undergoing neoadjuvant chemotherapy

6) Patients with immunodeficiency

7) Patients undergoing palliative surgery

8) Patients undergoing emergency surgery

　　　9) Otherwise judged by the investigator as unsuitable for enrollment

**9. Background and scientific rationale for the study (rationale and validity of the study)**

　　　While there are numerous reports on the usefulness of epidural analgesia after open surgery, the usefulness of epidural analgesia after laparoscopic surgery is not settled. Epidural analgesia may be associated with the risk of serious complications such as haematoma, abscess and nerve damage. Furthermore, adverse events such as postoperative hypotension and urinary retention have been reported with epidural analgesia, which, while effective for pain relief, may lead to delayed weaning and may not lead to a true early postoperative recovery. Furthermore, with regard to analgesia after laparoscopic colorectal resection for colorectal cancer, it has been reported that epidural analgesia rather delays recovery.^8, 11)^

　　　On the other hand, there have been few studies on analgesia after laparoscopic gastrectomy and no reports on the usefulness of epidural analgesia. If iv-PCA proves to be non-inferior to epidural analgesia (EDA) in postoperative analgesia for laparoscopic gastrectomy, it could provide safer and more effective postoperative analgesia and lead to a faster recovery for gastric cancer surgery patients.

The target number of cases and the rationale for setting the target number of cases are shown in section 6.

**10. Procedures for obtaining informed consent.**

The explanatory and consent documents approved by the Clinical Research Review Expert Committee will be given to the patients, sufficient explanation will be given in writing and orally, and the patients' free-will consent will be obtained in writing. When information on efficacy, safety, etc. that may affect the patient's consent is obtained, or when changes are made to the implementation plan, etc. that may affect the patient's consent, information is promptly provided to the patient, the patient's intention to participate or not in the research, etc. is confirmed in advance, and the patient's consent to the clinical research, etc. is obtained in advance. The patient's consent shall be obtained by revising the explanation and consent documents, etc., after obtaining the approval of the expert committee for the review of clinical research.

The consent explanatory document shall include the following.

1. The research has been authorised by the head of the research organisation to carry out the research concerned.

2. The name of the research organisation and the name of the principal investigator.

3. Purpose and significance of the research.

4. Methods of the research and duration.

5. Reasons for selection as research subjects.

6. Burdens and anticipated risks and benefits arising from participation in research.

7. Subjects may withdraw their consent at any time, even if they have given their consent for the research to be carried out or continued.

8. Research subjects by refusing to consent to research being conducted or continued or by withdrawing consent, that they will not be treated unfavourably.

9. Methods of disclosing information on research.

10. Access to or inspection of the research protocol and materials on research methods at the request of the research subject.

11. Handling of personal data and other information.

12. Methods of storage and disposal of samples and information.

13. Sources of funding for research, etc., conflicts of interest related to researcher, research institutions and earnings of individuals, etc.

14. Responding to consultations with research subjects and other relevant parties.

15. If there is any financial burden or gratuity to the research subjects, etc., a statement the details of such burden or gratuity.

16. In the case of research involving medical treatment beyond normal practice, matters relating to other treatment methods, etc.

17. In the case of research involving medical treatment that goes beyond normal medical treatment, medical treatment after the research has been carried out on the research subjects.

18. If, as a result of the conduct of the research, important findings concerning the health of the research subjects, genetic characteristics that can be passed on to their offspring, etc. How to handle research results.

19. In the case of research involving invasive procedures, whether or not there is compensation for health damage caused by the research and the nature of such compensation.

20. Samples and information obtained from research subjects

21. The person engaged in monitoring, the person engaged in auditing and the hospital ethics committee shall have access to the samples and information concerning the research subject concerned to extent necessary.

22. Intellectual property rights, ownership rights

**11. Handling of personal data and other information**

All researchers involved in the study will conduct it in compliance with the Declaration of Helsinki and the Ethical Guidelines for Medical Research Involving Human Subjects.

When handling samples and other materials related to the conduct of research, manage them with a research-specific subject code and give due consideration to the protection of the confidentiality of the research subjects. When publishing the results of research, information that can identify research subjects should not be included. In addition, the samples, etc. of research subjects obtained in the research shall not be used for any purpose other than the purpose of the research.

**12. Burdens, anticipated risks, and benefits to the research subjects**

**expected benefits.**

There will be no direct benefit to the study subjects from participating in this study. Study results may contribute to future medical advances regarding optimal analgesia after laparoscopic gastrectomy.

**anticipated disadvantages (adverse effects)**

Both epidural analgesia and intravenous patient-controlled analgesia are currently commonly used postoperative analgesia and are covered by insurance, although epidural analgesia is a more invasive procedure and carries the potential risk of serious complications such as haematoma, abscess and nerve damage. The frequency of serious complications is considered to be extremely rare, reported to be around 1 in 150,000-190,000 cases. In addition, epidural analgesia is a specialised procedure, which may increase the burden on study subjects compared to intravenous patient-controlled analgesia due to the procedure fees associated with epidural analgesia tube placement. As epidural analgesia is currently the standard of care in laparotomy, patient-controlled analgesia may result in increased post-operative pain compared to epidural analgesia.

**treatment of research subjects in the event of an adverse event**

If an adverse event is observed, the physician in charge should immediately take appropriate action and document it in the medical record and in the adverse event report form. The research subject should also be informed if the respective analgesic method is discontinued or if treatment for the adverse event becomes necessary.

**changes to research protocols etc.**

Collect and review information necessary for the safe conduct of clinical research. If new safety information, etc. is obtained, the research protocol and consent explanation document shall be changed as necessary. Any changes or revisions to the research protocol or consent explanation document must be approved in advance by the Clinical Research Review Expert Committee.

**discontinuation criteria for individual research subjects**

　[Action taken when research is discontinued].

If the principal investigator or a research assistant decides that it is not possible to continue the research on an individual research subject for any of the following reasons, the research on that research subject will be discontinued. In such cases, the reason for discontinuation will be explained to the research subjects, if necessary. The treatment of the research subject after discontinuation will be handled in good faith so as not to disadvantage the research subject.

[Discontinuation criteria].

(i) If the research subject offers to withdraw from participation in the research or withdraws their consent.

(ii) If the entire study is discontinued.

(iii) If the doctor in charge considers it appropriate to discontinue the study due to complications or other reasons.

**13. Methods of storage and destruction of samples and information**

The principal investigators and research assistants will store essential documents related to the conduct of research and other activities in the Clinical Research Building, Okayama University Department of Gastroenterological Surgery, 8th Floor, Clinical Research Building, Okayama University for five years after the completion of the research, after which they are destroyed.

**14. The sources of funding for the research, conflicts of interest related to the research for the duration of the research and the individual's earnings.**

There are no conflict of interest issues.

**15. Methods of disclosing information on research**

The study will be registered in the public database set up by the Union of Heads of National University Hospitals (UMIN). The results obtained from this study will also be presented at the respective relevant conferences and published as articles in specialised journals. In all cases, the published results will only be statistically processed and no personal information of the study subjects will be disclosed.

**16. Consultation and other responses from research subjects and others**

Principal investigator: Toshiyoshi Fujiwara

Dept of Gastroenterological Surgery, Okayama University Graduate School of Medicine, Dentistry and Pharmaceutical Sciences

2-5-1, Shikata-cho, Kita-ku, Okayama City, Okayama Prefecture, 700-8558

Tel: +81862357257

Fax: +81862218775

E-mail: toshi_f@md.okayama-u.ac.jp

　　　Research Secretariat: Satoruhi

Dept of Gastroenterological Surgery, Okayama University Graduate School of Medicine, Dentistry and Pharmaceutical Sciences

2-5-1, Shikata-cho, Kita-ku, Okayama City, Okayama Prefecture, 700-8558

Tel: +81862357257

Fax: +81862218775

E-mail: shinkuro@okayama-u.ac.jp

**17. What to do in the event of a serious adverse event**

In the event of a serious adverse event (failure), the principal investigator shall take the necessary action and follow the Standard Operating Procedures for Serious Adverse Events and Failures in Medical Departments at Okayama University.

Information on such adverse events is shared with researchers and others involved in the conduct of the study.

**18. Compensation for health hazards**

In the unlikely event that a research subject suffers a health hazard as a result of participation in this study, he or she may apply for compensation using the Adverse Reactions to Drugs Relief System. If a health hazard occurs to a research subject, appropriate measures will be taken and the necessary treatment, such as examination and treatment, will be provided within the research subject's insurance cover.

**19. Termination, discontinuation or suspension of research**

**termination of the study**

At the end of the research, the principal investigator will promptly submit a report on the completion of the research to the head of the research organisation.

**discontinuation or suspension of research**

The principal investigator will consider whether or not to continue the implementation of the research if any of the following apply When a decision is made to discontinue or suspend the research, it shall be promptly reported in writing to the head of the research organisation (or the head of the respective medical institution), together with the reasons for the decision.

**20. The possibility of using the samples and information obtained in this study for future research**

If the stored material is to be used for another purpose, a new application should be submitted to the University's Clinical Research Review Expert Committee for approval.

**21. Monitoring and auditing systems**

**monitoring**

As a rule, regular monitoring is carried out once a year for the purpose of ensuring that the study is conducted safely and in accordance with the research protocol, that consent forms are obtained and stored, and that data are collected accurately. Monitoring is centralised monitoring based on data from completed case record forms (CRFs) that are collected at the research office. Monitoring items are carried out in accordance with the monitoring plan.

**protocol deviations**

1) Violation: a deviation from the protocol rules that is clinically inappropriate and one of the following items constitutes a 'violation'

(i) Affects primary/secondary endpoints

(ii) The doctor in charge of the case is responsible for it.

(iii) Intentional or systematic

(iv) The degree of danger or deviation is extreme.

2) Deviations: deviations that do not fall into the category of either violation or tolerance. If a number of specific deviations are found, they should be noted in the publication of the research results.

3) Tolerance: to be decided in advance by the principal investigator/research office.
